# Biomarker-concordant Steroid use in Hospitalized Patients with Community-acquired Pneumonia: A Prospective Cohort Study

**DOI:** 10.2174/0118743064482683260618204843

**Published:** 2026-06-29

**Authors:** Claudia Gyimah, Felix Wireko, Amy Amsbaugh, Lori Dahring, Mohamad El Labban, Susie Kuhns, Alpha Amadi, Yewande Odeyemi, Aysun Tekin

**Affiliations:** 1 Division of Pulmonary and Critical Care Medicine, Department of Internal Medicine, Mayo Clinic, Rochester, MN, United States of America; 2 Anesthesia Clinical Research Unit, Mayo Clinic, Rochester, MN, , United States of America; 3 Department of Internal Medicine, Mayo Clinic Health System, Mankato, MN, United States of America; 4 Clinical Research Internship Study Program, Mayo Clinic, Rochester, MN, United States of America; 5 Department of Nursing, Mayo Clinic, Rochester, MN, United States of America

**Keywords:** Biomarker-guided therapy, Community-acquired pneumonia, Corticosteroids, Inflammatory biomarkers, Neutrophil-to-lymphocyte ratio, Observational cohort study, Prognosis

## Abstract

**Introduction:**

The neutrophil-lymphocyte ratio (NLR) is an emerging inflammatory biomarker in community-acquired pneumonia (CAP) with potential prognostic implications. Biomarker-concordant corticosteroid dosing strategies based on other inflammatory markers have been found to be associated with improved clinical outcomes. The NLR is a readily available and affordable biomarker and could help guide corticosteroid use in CAP. Our goal is to evaluate the association between NLR-concordant corticosteroid prescription and patient outcomes.

**Methods:**

Single-center prospective cohort study of adults with CAP who were admitted to the Mayo Clinic in Rochester, Minnesota, between December 10, 2023, and March 11, 2025. Patients with available NLR within 24 hours of admission were evaluated. Those who received steroids after 24 hours of admission, stayed in the hospital shorter than 24 hours, and were not yet discharged were excluded. Steroid use was classified as 'biomarker-concordant' if given when NLR > 12 or withheld when NLR <= 12, and 'biomarker-discordant' otherwise. All-cause mortality was the primary outcome.

**Results:**

Of 545 admissions with CAP, 465 were included. Steroid use was biomarker-discordant in 222 (47.7%) patients and biomarker-concordant in 243 (52.3%) patients. Systemic corticosteroid use within 24 hours was more common in the discordant group (127 (57.2%) *versus* 77 (31.7%)). After adjusting for the pneumonia severity index, there were no significant differences in clinical outcomes between groups including in-hospital mortality (odds ratio [95% C.I.] = 1.120 [0.463, 2.706]), 30 – day mortality (hazard ratio [95% C.I.] = 1.423 [0.747, 2.710]), oxygen-free days (estimate [95% C.I.] = 0.61 [–0.655, 1.879), or hospital-free days (estimate [95% C.I.] 0.222 [–0.943, 1.386]) between biomarker-discordant *versus* concordant corticosteroid use.

**Discussion:**

Despite growing interest in biomarker-guided corticosteroid strategies, NLR-based concordance was not associated with clinical outcomes in this cohort, suggesting that its role may be limited to risk stratification rather than treatment guidance.

**Conclusions:**

In this observational cohort of hospitalized CAP patients, a biomarker-concordant corticosteroid treatment strategy based on the NLR was not associated with improved clinical outcomes.

## 1 INTRODUCTION

Community-acquired pneumonia (CAP) continues to be a leading cause of hospitalizations and mortality. In the United States, it is estimated that CAP results in approximately 740,000 hospitalizations and 41,000 deaths annually, with mortality as high as 26.8% for hospitalized patients aged 60 years or older with comorbidities [1]. Several tools have been validated for risk stratification and prognostication in CAP including the pneumonia severity index (PSI), Confusion, Urea, Respiratory rate and Blood pressure (CURB), Confusion, Urea, Respiratory rate and Blood pressure, aged 65 and older (CURB-65) scores, and the American Thoracic Society/ Infectious Disease Society of America (ATS/IDSA) guidelines, and these continue to be used to assess severity and guide management [2-5]. Biomarkers like procalcitonin, C- reactive protein (CRP), and interleukin-6 have been used to predict disease severity and treatment response [6]. However, their use has been limited due to their poor specificity [6].

The Neutrophil-Lymphocyte ratio (NLR) is a marker of systemic inflammatory response and is calculated by dividing the absolute neutrophil count by the absolute lymphocyte count from a complete blood count [7]. It is an emerging prognostic biomarker in several conditions, including sepsis, pneumonia, COVID-19, and cancer, and has been reported to correlate independently with mortality in the general population and in patients with pneumonia, influenza, chronic lower respiratory diseases, heart disease, and kidney disease [7, 8]. The NLR was also found to correlate with mortality after CAP more strongly than other pneumonia scoring systems and biomarkers, including the PSI, CURB-65, white blood cell count, and CRP [9, 10]. However, these results have been inconsistent, with some studies showing no additional benefit in prognostication [11]. In a single-center retrospective analysis of hospitalized CAP patients, the NLR was not found to be superior to PSI for in- hospital or 6-month mortality prediction [11]. More recent observational studies and meta-analyses have further confirmed that elevated NLR is associated with higher short-term mortality in CAP and may modestly improve risk stratification when added to existing severity scores [12, 13]. Prospective studies are needed to further evaluate the prognostic role of NLR in CAP.

Since antibiotics alone fall short in curbing excessive systemic inflammation in CAP, there has been a growing interest in anti-inflammatory therapies, specifically corticosteroids [14-20]. Despite multiple studies, the use of corticosteroids in CAP remains undefined, with varying guideline recommendations. This is potentially due to significant heterogeneity in past randomized clinical trials, resulting in limited optimal patient selection strategies that could predict corticosteroid responsiveness on admission. Because the benefit of corticosteroids is mediated primarily through their anti-inflammatory effects [14, 21], studies have evaluated the role of inflammatory markers to guide their use. For example, in two retrospective studies, a biomarker-concordant corticosteroid use strategy, utilizing CRP as an inflammatory marker, was associated with improved clinical outcomes in CAP and COVID- 19 pneumonia [11, 17, 22]. Additionally, a single center randomized controlled trial (RCT), found that an individualized CRP-guided corticosteroid dosing approach was associated with increased oxygen-free days, hospital-free days, and lower cumulative corticosteroid exposure in COVID- 19 pneumonia. More recently, CRP was identified as a major effect modifier in a subgroup analysis of an RCT [23] and in studies utilizing individual patient data from multiple RCTs to estimate the corticosteroid effect in CAP [20]. Although these studies suggest that CRP may predict corticosteroid responsiveness, it is still not routinely used or recommended in clinical practice.

While the NLR is a documented predictor of corticosteroid responsiveness in COVID-19 pneumonia [24], its relevance in CAP remains uncertain [25], with existing evidence largely supporting only its prognostic role [9]. As a biomarker derived from routine complete blood count, the NLR is a more readily available inflammatory marker than others in hospitalized patients and could potentially guide corticosteroid use in CAP. Our study aims to evaluate the frequency of prescribing corticosteroids in an NLR-concordant manner and its association with CAP outcomes.

## 2 METHODS

This study is reported in accordance with the STROBE (Strengthening the Reporting of Observational Studies in Epidemiology) guidelines, and the completed checklist is provided. The Mayo Clinic Institutional Review Board reviewed this study and determined it to be exempt (#25-003789). The requirement for informed consent was waived. The study was conducted in accordance with the ethical principles outlined in the Declaration of Helsinki. An infographic summarizing the study design and primary outcomes is provided in Fig. (**1**).

### 2.1 Design, Setting, and Participants

This is a cohort study conducted on the dataset prospectively collected within the scope of the CAP Registry project.

#### 2.1.1 CAP Registry

The CAP Registry was initiated at Mayo Clinic Rochester in December 2023, and it includes all adult patients hospitalized with CAP. Each weekday morning, a query of the electronic health record system (Epic Reporting Workbench [15]) generates a list of patients admitted within the prior 24 hours whose admission diagnosis suggests pneumonia. Eligibility is then confirmed through manual review of clinical details against prespecified criteria.

The inclusion criteria are:

-Adult patients (≥18 years) hospitalized for acute (≤14 days) onset of symptoms (cough, sputum production, or dyspnea).

-Radiographic evidence of pneumonia.

The exclusion criteria are:

-Pneumonia diagnosed after 48 hours of admission or after 48 hours of intubation,

-Absence of Minnesota research authorization.

For this study, patients registered between December 10, 2023, and March 11, 2025, were screened. Patients still hospitalized at the time of data analysis, admissions shorter than 24 hours, and those who received corticosteroids only after the first day of admission were excluded (Fig. **2**). The study population consisted of all consecutive adult patients hospitalized with CAP and enrolled in the CAP Registry during the study period who met eligibility criteria. Given the observational nature of the study and the use of a complete eligible cohort, no formal sample size or power calculation was performed. The sample size and demographic characteristics, including sex distribution, were determined by the underlying population of consecutively enrolled patients in the registry.

### 2.2 Variable Definitions

The exposures of interest, *i.e.*, NLR and administration of systemic corticosteroids, were assessed within 24 hours of admission. Laboratory results are reported in 10^9^/L, with institutional reference ranges of 3.4–9.6 for total white blood cell count, 1.56–6.45 for neutrophils, and 0.95–3.07 for lymphocytes. For each patient, the earliest available neutrophil and lymphocyte counts within the first 24 hours were recorded as continuous variables and used to calculate the NLR [11]. Based on prior evidence, a threshold of 12 was used to categorize patients (≤12 *vs*. >12) [10]. Systemic corticosteroid therapy was defined as intravenous, intramuscular, or oral administration. Inhaled or topical corticosteroids alone were not considered systemic therapy. The flowchart for the identification of patients and patient distribution is shown in Fig. (**2**).

To quantify illness severity, the PSI was calculated based on clinical characteristics and laboratory results within the first day of admission [16]. For statistical analyses, the raw PSI score was modeled as a continuous variable to retain precision, rather than stratifying into classes. Because the PSI already incorporates baseline factors such as age, sex, and comorbidities, these were not included separately.

### 2.3 Outcomes

The primary outcome was all-cause mortality, assessed at hospital discharge and at 30 days post-admission. Secondary outcomes were the requirement for admission to the intensive care unit (ICU), requirement for vasopressors, hospital-free days (HFD), oxygen-free days, and invasive mechanical ventilation (IMV)-free days. We calculated HFD as the number of days alive and out of the hospital within 28 days of admission, with a score of 0 assigned to those who died during the hospitalization or remained hospitalized for longer than 28 days [19]. Oxygen- and IMV-free days were defined similarly. To ensure that all exposures clearly preceded the outcomes measurement, patients who were admitted to the ICU or were receiving vasopressor support during the first 24 hours were excluded from the corresponding analyses. This approach maintained a strict temporal separation between exposure assessment and outcome measurement. Because our primary exposures were defined within the initial 24-hour window, including patients who experienced the outcome during that same period would have introduced ambiguity about whether the exposure could have plausibly influenced the event. The applied exclusions were specific to the outcome, and the number of patients available for evaluating those outcomes was reported in the tables.

### 2.4 Statistical Analysis

Continuous variables were summarized as medians with interquartile ranges, while categorical variables were expressed as frequencies and percentages. For unadjusted comparisons, the chi-square test was used for categorical data, and the Mann–Whitney U test for continuous variables.

Survival was assessed at 30 days using Kaplan–Meier curves with log-rank testing. Multivariable analyses incorporated the raw PSI score to adjust for baseline severity *via* multivariable logistic regression, linear regression, and Cox proportional hazards models. Patients with missing key exposure or outcome data (*e.g.*, missing NLR values or discharge data) were excluded from the analysis; no additional imputation was performed, and analyses were conducted using complete-case data. Results were presented as odds ratios (OR), hazard ratios (HR), *p*-values, estimates, and 95% confidence intervals (C.I.). The discordant group was used as the reference. To explore whether the impact of corticosteroids differed by inflammatory status, we stratified patients into two groups based on their NLR levels (≤ 12 *vs*. > 12) and analyzed outcomes within each group. Calculations were performed using IBM SPSS v27.0 (Statistical Package for Social Sciences, USA) statistical software program, and a two-sided *p*-value of 0.05 was deemed significant.

## 3 RESULTS

**Fig. (1):**
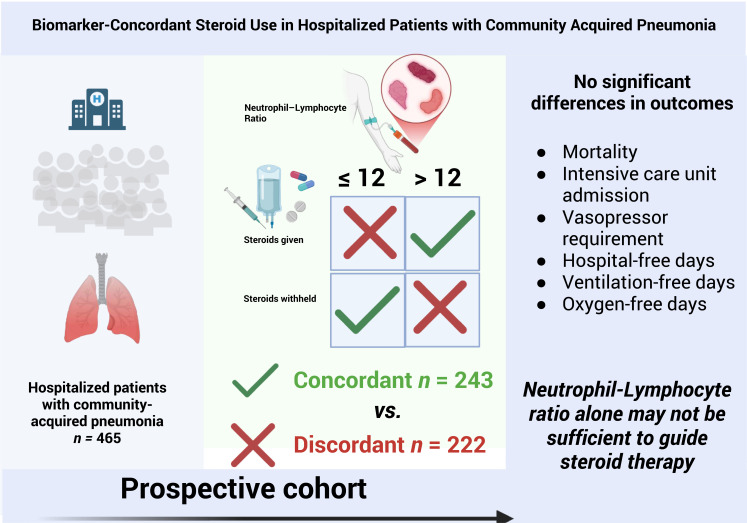
Infographic summarizing the study design and primary outcomes.

**Fig. (2):**
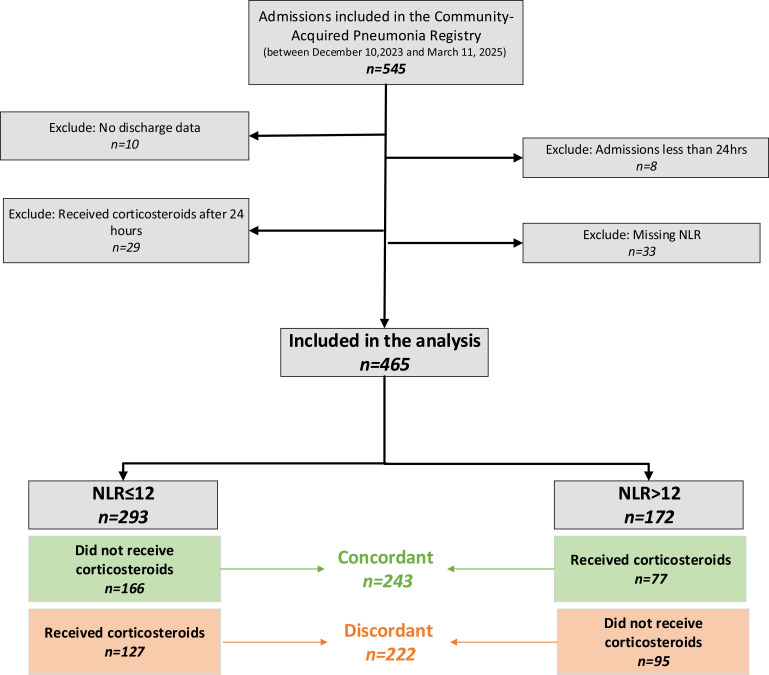
Flowchart for the identification of patients. Admissions to the community-acquired pneumonia registry were screened, with exclusions applied for admissions <24 hours, missing discharge data, corticosteroid administration after 24 hours, and missing NLR values. NLR: Neutrophil/lymphocyte ratio.

A total of 545 patients were included in the CAP Registry between December 10, 2023, and March 11, 2025 (Fig. **1**), of whom 465 had an NLR measured within 24 hours of admission and were included in the analysis. Among these, 222 (47.7%) were classified as the biomarker-discordant group and 243 (52.3%) as the biomarker-concordant group. Out of the concordant group, 77 patients received corticosteroids, and 166 did not, while in the discordant group, 127 received corticosteroids and 95 did not (31.7% *vs*. 57.2%, *p*<0.001).

Baseline demographic and clinical characteristics were similar between groups (Table **1**). The median age was 71 years in both groups, and 55.9% of the overall cohort were male. The racial and ethnic distributions were broadly comparable and were predominantly non-Hispanic whites, though comparative tests could not be performed due to sparse distribution. Comorbidity burden, assessed by the Charlson Comorbidity Index, was high across the cohort, with a median of 10 (7–12) without significant group differences.

The median NLR was 9.5 (5.09- 16.61) and was higher in the discordant group compared with the concordant group, 10.49 (5.58–18.34) *vs*. 8.69 (4.96–15.55), although not statistically significant (*p*=0.061). Other early clinical interventions—including vasopressor initiation and ICU admission within 24 hours—did not differ between groups. Pneumonia severity was comparable, as reflected by similar PSI raw scores and risk class distributions.

### 3.1 Outcomes: Primary and Secondary

**Fig. (3):**
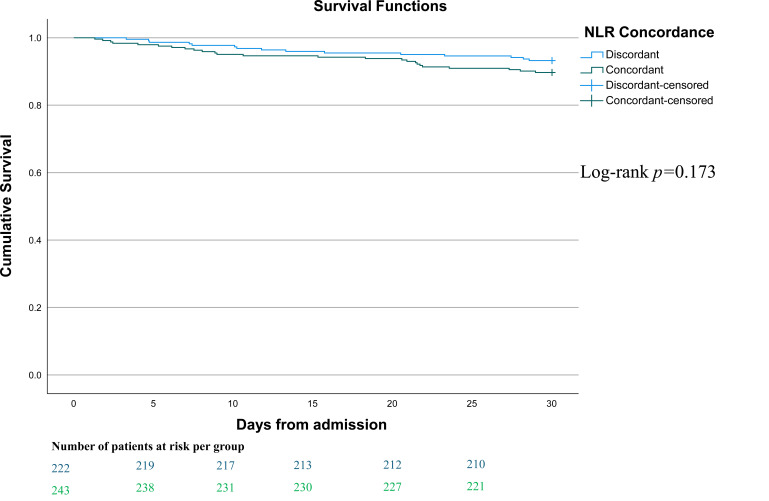
Kaplan–Meier survival curves illustrating 30-day mortality according to study groups. The x-axis represents time in days from admission, and the y-axis represents survival probability. Numbers at risk for each group are displayed below the x-axis at specified time points.

Hospital mortality did not differ significantly between discordant and concordant treatment groups, either before (4.5% *vs*. 6.2%, *p*=0.426) or after adjustment for PSI score (adjusted odds ratio [aOR], 1.12; 95% CI, 0.46–2.71; *p*=0.802). The 30-day mortality rate was numerically higher in the concordant group, though this difference was not statistically significant (10.3% *vs*. 6.8%, *p*=0.173) (Fig. **3**). Multivariable Cox regression showed no significant association between biomarker-concordant treatment and 30-day mortality after adjusting for the PSI score (adjusted hazard ratio, 1.42; 95% CI, 0.75–2.71; *p*=0.283) (Table **2**).

Rates of ICU admission (4.2%) and vasopressor requirement (5.2%) during hospitalization were low overall and remained similar between groups in both univariable and multivariable models. Functional recovery outcomes were also similarly comparable. Hospital-free days and IMV–free days did not differ between groups in either unadjusted or adjusted analyses. The concordant group had slightly more oxygen-free days in univariable comparison (26.51 *vs*. 25.67 days, *p*=0.048), but this difference was no longer significant after adjustment (0.61; 95% CI, –0.66-1.88; *p*=0.343) (Table **2**).

## 4 DISCUSSION

In this prospective cohort study of adults hospitalized with CAP, we found that using the NLR as a predictive biomarker to guide corticosteroid use was not associated with improvements in mortality, ICU admission, vasopressor requirement, hospital-free days, oxygen-free days, or IMV-free days. Additionally, about half of patients received corticosteroids in a discordant manner with their inflammatory phenotype, yet adjusted analyses demonstrated no meaningful differences in clinical outcomes between groups. Our findings suggest that NLR-based steroid guidance does not influence clinical outcomes in hospitalized CAP patients.

The use of biomarker-guided strategies for corticosteroid use in CAP offers an individualized approach that could potentially maximize the benefits of adjunct corticosteroids while minimizing their adverse effects [14]. Current evidence supports the use of CRP as a predictor for corticosteroid responsiveness in CAP, but unfortunately, CRP is not routinely measured on admission [14, 18, 20, 21, 26, 27]. A recent review of biomarker-guided corticosteroid strategies in pneumonia similarly concludes that CRP-based selection appears most promising to date, whereas prospective NLR-guided approaches are still lacking [28]. Given that corticosteroids work primarily by modulating dysregulated systemic inflammation, the reported benefits are biologically plausible [14, 21]. They also reflect the central role of hyperinflammation in severe respiratory infections and the potential for biomarkers to help identify patients most likely to respond to steroid modulation [21].

The NLR has been associated with predicting disease severity and mortality in CAP, sepsis, COVID-19, and other inflammatory conditions [25, 29, 30]. Our study adds prospective evidence to a growing body of literature on the potential role of inflammatory biomarkers in guiding corticosteroid use in pneumonia. The role of the NLR in predicting corticosteroid responsiveness is documented in COVID-19 pneumonia [24], but its utility in CAP remains uncertain [25] as the available evidence primarily supports its prognostic utility in these settings [9]. Other composite indices that incorporate neutrophil parameters, such as the neutrophil percentage-to-albumin ratio or platelet-to-neutrophil ratio, may offer stronger discrimination of risk than NLR alone in glucocorticoid-treated patients [31]. There is concern that the NLR may not sufficiently identify the inflammatory phenotype most responsive to corticosteroids, particularly when compared to CRP, which reflects a more specific inflammatory pathway [32]. This was reflected in our study, where NLR-based steroid initiation did not influence clinical outcomes in the CAP cohort.

Several factors may explain why an NLR-based concordance strategy did not translate into outcome differences in our cohort. First, the NLR may reflect overall disease severity rather than treatment responsiveness. Although it correlates with inflammation, it is an indirect marker influenced by physiological stress, comorbidities, and non-infectious conditions [7] and may be a less sensitive tool for identifying corticosteroid-responsive inflammatory states in CAP. Conversely, CRP has been identified as a stronger predictor of corticosteroid responsiveness in CAP [20]. Second, the level of steroid exposure in both groups in our study cohort may have attenuated observable differences. More than half of the discordant group received steroids, and about a third of the concordant group also received steroids, regardless of their inflammatory phenotype. This reflects the variability in steroid prescription among clinicians, who are guided more by their clinical gestalt than by biomarker thresholds. It also underscores the heterogeneity in guideline recommendations and the absence of validated biomarker-guided strategies for steroid initiation [33]. Third, although steroid responsiveness is more pronounced in severe CAP, where inflammatory dysregulation is greatest, the overall severity of illness in our study population was moderate, as evidenced by relatively low rates of ICU admission, vasopressor use, and IMV requirement. The broad spectrum of severity in our cohort may have diluted the treatment effect even within biomarker strata, as the current evidence for corticosteroid use shows benefit in only severe CAP but not mild to moderate CAP [14]. Lastly, our use of a fixed NLR threshold [23], based on prior prognostic literature [10], may not reflect the optimal cut-off for identifying steroid-responsive phenotypes.

The strengths of this study include the prospective design, data collection within a structured CAP registry, standardized exposure assessments within 24 hours of admission, and adjustment for illness severity using the continuous PSI score. Also, the use of only early biomarkers and treatment exposures minimizes bias related to clinical deterioration preceding steroid initiation. Additionally, the study reflects real-world clinical practice, enhancing external generalizability.

Our study has several limitations worth mentioning. Even though it reflects real-world clinical practice, it is still a single-center study, and practice patterns may not apply to settings with differing patient profiles or prescribing patterns. Although the data were prospectively collected, the observational, registry-based design is subject to inherent limitations. Furthermore, the racial and ethnic homogeneity of our cohort may limit the generalizability of our findings to more diverse populations, where differences in CAP epidemiology and inflammatory responses could influence both biomarker performance and treatment effects. Although we captured early steroid exposure, we did not quantify cumulative steroid dose, duration, or timing beyond the first 24 hours, which may influence treatment effects. Similarly, we limited the evaluation of NLR to values obtained within the first 24 hours of admission and did not assess serial or trajectory-based NLR measurements, which may have led to an underestimation of the biomarker’s utility. Also, the modest event rates, especially for ICU admissions and mortality, may have potentially limited the power to detect small but clinically meaningful differences. Finally, the use of an NLR threshold of 12 may not represent the optimal cut-off for steroid responsiveness. Alternative thresholds, or models that treat NLR as a semi-continuous variable, may offer improved discriminatory utility and provide additional insights into dose–response relationships, and this represents an important area for future investigation.

## CONCLUSION

Among hospitalized adults with CAP, an NLR-based biomarker-concordant corticosteroid strategy was not associated with improvements in clinical outcomes. These findings suggest that the NLR may not be sufficient as a standalone biomarker for guiding corticosteroid therapy in this population. Future studies should explore alternative biomarkers, biomarker combinations, or dynamic inflammatory trajectories to accurately identify patients most likely to benefit from corticosteroid therapy.

## Figures and Tables

**Table 1:** Demographics and clinical characteristics of patients.

**Variables**	**Total (*n*=465)**	Discordant* (*n=*222)	Concordant* (*n*=243)	***p***
**Age, median (IQR)**	71 (61, 81)	71 (61, 81)	71 (61, 80)	0.798
**Sex, no. (%)**				
**Male**	260 (55.9)	130 (58.6)	130 (53.5)	0.272
**Race, no. (%)**				NA**
**White**	430 (92.5)	207 (93.2)	223 (91.8)	
**African American**	16 (3.4)	6 (2.7)	10 (4.1)	
**Asian**	12 (2.6)	3 (1.4)	9 (3.7)	
**Other or unknown**	7 (1.5)	6 (2.7)	1 (0.4)	
**Ethnicity, no. (%)**				NA**
**Non-Hispanic**	439 (94.4)	210 (94.6)	229 (94.2)	
**Hispanic**	19 (4.1)	8 (3.6)	11 (4.5)	
**Other or unknown**	7 (1.5)	4 (1.8)	3 (1.2)	
**Charlson Comorbidity Index, median (IQR)**	10 (7, 12)	10 (7, 12)	10 (7, 13)	0.729
**Neutrophil/Lymphocyte ratio, median (IQR)**	9.50 (5.09, 16.61)	10.49 (5.58, 18.34)	8.69 (4.96, 15.55)	0.061
**Received vasopressors within 24 hours of admission, no. (%)**	45 (9.7)	17 (7.7)	28 (11.5)	0.159
**Received systemic steroids within 24 hours of admission, no. (%)**	204 (43.9)	127 (57.2)	77 (31.7)	<0.001
**Admitted to the intensive care unit within 24 hours of admission, no. (%)**	108 (23.2)	55 (24.8)	53 (21.8)	0.450
**Pneumonia severity index, raw score, median (IQR)**	138 (111, 165)	132 (112, 163)	141 (109, 167)	0.606
**Pneumonia severity index risk class, no. (%)**				0.269
**Class I**	7 (1.5)	1 (0.5)	6 (2.5)	
**Class II**	25 (5.4)	11 (5.0)	14 (5.8)	
**Class III**	27 (5.8)	15 (6.8)	12 (4.9)	
**Class IV**	143 (30.8)	74 (33.3)	69 (28.4)	
**Class V**	263 (56.6)	121 (54.5)	142 (58.4)	

**Note:** * Corticosteroid use was defined as either biomarker concordant: steroids given when Neutrophil/Lymphocyte ratio (NLR) level is >12 or withheld when NLR level is <=12, or biomarker-discordant: steroids given when NLR level is <=12 or withheld when NLR level is >12. ** *P*-values were not calculated because of the sparse distribution. **Abbreviation:** IQR: interquartile range.

**Table 2:** Primary and secondary outcomes based on concordance.

Variables	Total (*n*=465)	Discordant* (*n=*222)	Concordant* (*n*=243)	Univariable Analysis	Multivariable Analysis**	
				***P*-value**	**Odds ratio (95% CI)**	***P*-value**
Hospital mortality, no. (%)	25 (5.4)	10 (4.5)	15 (6.2)	0.426	1.12 (0.46, 2.71)	0.802
Required intensive care unit admission, no. (%) (n=357)***	15 (4.2)	9 (5.4)	6 (3.2)	0.294	0.59 (0.2, 1.7)	0.327
Required vasopressor treatment, no. (%) (n=420) ***	22 (5.2)	11 (5.4)	11 (5.1)	0.909	0.95 (0.4, 2.25)	0.906
				***P*-value**	**Hazard ratio (95% CI)**	***P*-value**
30-day mortality, no. (%)	40 (8.6)	15 (6.8)	25 (10.3)	0.173	1.42 (0.75, 2.71)	0.283
				***P*-value**	**Estimate (95% CI)**	***P*-value**
Hospital free days, median (IQR)	23.99 (20.72, 25.75)	24.00 (20.73, 25.51)	23.99 (20.49, 25.81)	0.615	0.22 (-0.94, 1.39)	0.709
Oxygen-free days, median (IQR)	26.2 (23.5, 27.8)	25.67 (23.25–27.63)	26.51 (23.94–27.94)	0.048	0.61 (-0.66, 1.88)	0.343
Invasive mechanical ventilation free days, median (IQR)	28.00 (28.00–28.00)	28.00 (28.00–28.00)	28.00 (28.00–28.00)	0.554	-0.35 (-1.58, 0.87)	0.573

**Note:** * Corticosteroid use was defined as either biomarker concordant: steroids given when Neutrophil/Lymphocyte ratio (NLR) level is >12 or withheld when NLR level is <=12, or biomarker-discordant: steroids given when NLR level is <=12 or withheld when NLR level is >12. *Data were analyzed using multivariable regression models adjusting for pneumonia severity score (raw). The discordant group was the reference. **Abbreviation:** CI: Confidence interval.

## Data Availability

The data and supportive information are available within the article. Additional data may be available upon reasonable request to the corresponding author, subject to applicable regulations, institutional data sharing agreements, and ethical approvals.

## LIST OF ABBREVIATIONS

CAP: Community-acquired pneumonia
PSI: Pneumonia severity index
NLR: Neutrophil-Lymphocyte ratio
RCT: Randomized controlled trial

## STANDARDS OF REPORTING

STROBE guidelines were followed.
